# Comparison of Syngas-Fermenting *Clostridia* in Stirred-Tank Bioreactors and the Effects of Varying Syngas Impurities

**DOI:** 10.3390/microorganisms10040681

**Published:** 2022-03-22

**Authors:** Luis Oliveira, Anton Rückel, Lisa Nordgauer, Patric Schlumprecht, Elina Hutter, Dirk Weuster-Botz

**Affiliations:** Technical University of Munich, TUM School of Engineering and Design, Department of Energy and Process Engineering, Chair of Biochemical Engineering, 85748 Garching, Germany; luis.oliveira@tum.de (L.O.); anton.rueckel@tum.de (A.R.); lisa@nordgauer.eu (L.N.); patric.schlumprecht@gmail.com (P.S.); hutter.elina@web.de (E.H.)

**Keywords:** *Clostridium autoethanogenum*, *Clostridium ljungdahlii*, *Clostridium ragsdalei*, autotrophic alcohol production, syngas impurities, synthesis gas fermentation

## Abstract

In recent years, syngas fermentation has emerged as a promising means for the production of fuels and platform chemicals, with a variety of acetogens efficiently converting CO-rich gases to ethanol. However, the feasibility of syngas fermentation processes is related to the occurrence of syngas impurities such as NH_3_, H_2_S, and NO_X_. Therefore, the effects of defined additions of NH_4_^+^, H_2_S, and NO_3_^−^ were studied in autotrophic batch processes with *C. autoethanogenum*, *C. ljungdahlii*, and *C. ragsdalei* while applying continuously gassed stirred-tank bioreactors. Any initial addition of ammonium and nitrate curbed the cell growth of the *Clostridia* being studied and reduced the final alcohol concentrations. *C. ljungdahlii* showed the highest tolerance to ammonium and nitrate, whereas *C. ragsdalei* was even positively influenced by the presence of 0.1 g L^−1^ H_2_S. Quantitative goals for the purification of syngas were identified for each of the acetogens studied in the used experimental setup. Syngas purification should in particular focus on the NO_X_ impurities that caused the highest inhibiting effect and maintain the concentrations of NH_3_ and H_2_S within an acceptable range (e.g., NH_3_ < 4560 ppm and H_2_S < 108 ppm) in order to avoid inhibition through the accumulation of these impurities in the bioreactor.

## 1. Introduction

Syngas (synthesis gas) fermentation is the microbiological conversion of CO-, H_2_-, and CO_2_-rich gases to short chain fatty acids or alcohols. If renewable resources are the feedstock for the gasification to syngas, then the microbial production of commercially relevant chemicals demonstrates higher sustainability than fossil feedstock. The biochemical conversion of syngas represents an alternative to the thermochemical conversion to, e.g., ethanol, given the advantages of milder temperatures and pressures, flexibility to H_2_/CO ratios, and higher product selectivity [1].

Some members of the genus *Clostridium* are able to convert CO or CO_2_ and H_2_ to acetate and ethanol, along with other strain-specific products such as 2,3-butanediol, butanol, and hexanol [2]. These acetogens use the reductive acetyl-CoA pathway for autotrophic carbon fixation. Energy in the form of ATP is conserved by a membrane-bound ATP-synthase, which uses either H^+^ or Na^+^ gradients across the membrane [3].

*C. autoethanogenum*, *C. ljungdahlii*, and *C. ragsdalei* belong to the same clade [4], having a sequence similarity of more than 99% [5] and producing acetate, ethanol, and 2,3-butanediol from CO or CO_2_ and H_2_ [6]. The growth optima are at 37 °C with a pH optimum of pH 5.5–6.0 [7,8]. All three strains favor the use of CO over H_2_ and CO_2_, as theoretically proposed [9] and shown experimentally for *C. ljungdahlii* [10]. The batch process performances of *C. autoethanogenum* and *C. ljungdahlii* were studied in anaerobic flasks with 50 kPa, 45 kPa, and 5 kPa of CO, H_2_, and CO_2_, respectively. *C. autoethanogenum* and *C. ljungdahlii* achieved similar optical densities and acetate concentrations, whereas the highest ethanol and 2,3-butanediol concentrations were measured with *C. autoethanogenum* [7]. *C. ragsdalei* achieved lower optical densities, but it was able to produce more ethanol than acetate.

When generated from biogenic materials, syngas contains (along with the main gas components CO, CO_2_, H_2_, and N_2_) a variety of impurities, depending on the gasification process and feedstock. These might include H_2_S, NH_3_, COS, HCN, and NO_X_ [11]. The formation of these impurities can to some extent be controlled by the choice of the gasification conditions. Increasing the equivalence ratio (mass ratio between air and fuel in the gasification process) has been shown to increase the conversion of fuel bound nitrogen to ammonia and N_2_ but had no influence on the formation of HCN during the gasification of switchgrass [12].

It has already been demonstrated that syngas impurities accumulated in the aqueous fermentation broth may, depending on their concentrations, have an impact on the performance of the fermentation processes [13]. The addition of up to 29.4 mM sulfide, which corresponds to 1.0 g L^−1^ H_2_S, promoted autotrophic growth and alcohol formation in batch processes with *Clostridium carboxidivorans* in a continuously CO/CO_2_ gassed stirred-tank reactor [14]. However, even the addition of 1.9 mM sulfide (0.065 g L^−1^ H_2_S) slightly decreased cell dry weight (CDW) concentration and promoted ethanol production in anaerobic flasks with *C. ragsdalei* using artificial syngas as a substrate [15]. Sulfide inhibits anaerobic bacteria above a certain threshold and is associated with the undissociated H_2_S solved in water, which is membrane-permeable [16]. Once inside the cell, it can cause DNA damage and protein denaturation [17]. As a result, there are large differences between the effects of H_2_S on different anaerobic strains.

The addition of 93.5 mM NH_4_^+^ (5.00 g L^−1^ NH_4_Cl) promoted the autotrophic growth and alcohol formation of *C. carboxidivorans* in a continuously CO/CO_2_ gassed stirred-tank reactor [14], but the addition of 93.4 mM NH_4_^+^ (1.68 g L^−1^) showed no effect on the growth or product formation of *C. ragsdalei* in anaerobic flasks with a CO/CO_2_ atmosphere [18]. Ammonium has been shown to decrease the activity of hydrogenases and alcohol-dehydrogenases in acetogens [19], which catalyze the reactions for the supply of reduction equivalents from H_2_ or CO and for alcohol formation, respectively. Therefore, NH_4_^+^ concentrations above a certain threshold could be an obstacle in syngas fermentation processes with the concomitant supply of CO and H_2_/CO_2_.

Concentrations above 40 ppm nitric oxide in the syngas inhibited the autotrophic growth of *C. carboxidivorans* with H_2_/CO_2_, but this inhibition was reversible, and a complete inhibition of the hydrogenase activity was determined at 150 ppm NO [20].

Other nitrogen species also affect syngas fermentations with *Clostridia*. The addition of 0.1 g L^−1^ NaNO_3_ increased the lag phase of *C. carboxidivorans* to 30 h, but higher final CDW concentrations and a strong increase in butyrate concentrations was observed with artificial syngas in a stirred-tank bioreactor [14]. A total of 15 mM NaNO_3_ (1.275 g L^−1^) promoted the biomass growth of *C. ljungdahlii* in anaerobic flasks with H_2_ and CO_2_ as the gas phase, but growth was inhibited with CO and CO_2_ [21]. Moreover, the addition of nitrate reduced ethanol formation and promoted formate production. Substituting ammonium with nitrate on a molar basis led to higher optical densities in a chemostat at mean hydraulic residence times between 2 and 3.5 d using *C. ljungdahlii* with H_2_ and CO_2_ as the gas phase, but substituting ammonium with nitrate was associated with “stochastic metabolic crashes” [22]. *C. ljungdahlii* is able to reduce nitrate to ammonium, resulting in more than a doubling of the net ATP gain from H_2_ when compared to the production of acetate from H_2_ [21].

Information on the effect of syngas impurities on syngas fermentation processes is sparse, and the comparison between different strains is obscured by varying process designs and conditions [13]. Many of the published results are based on simple batch studies applying non-controlled anaerobic flasks with low gas amounts, low gas-liquid mass transfer rates, and low power input, but these conditions do not reflect scalable continuously gassed syngas fermentation processes and lack the quantitative analysis of gas consumption rates and, therefore, carbon balances. Our report represents an extensive comparison of fully controlled syngas fermentation processes with *C. autoethanogenum*, *C. ljungdahlii*, and *C. ragsdalei*, respectively, in stirred-tank bioreactors at defined power input, gassing rates, and identical medium compositions for studying the strain-specific effects of defined additions of ammonium, sulfide, and nitrate on batch process performance. The process performance of these *Clostridia* were compared to published results using *C. carboxidivorans* [14]. Since the syngas components investigated may be present as impurities in syngas, the quantitative criteria for syngas purification processes were identified. This study also provides insights into the choice of preferred strains respecting their individual capabilities of converting CO-rich syngas.

## 2. Materials and Methods

### 2.1. Microorganisms and Cultivation Media

*C. autoethanogenum* (DSM 10061), *C. ljungdahlii* (DSM 13528), and *C. ragsdalei* (DSM 15248) were obtained from the German Collection of Microorganisms and Cell Cultures (DSMZ, Braunschweig, Germany). Precultures were prepared in 500 mL flasks with a butyl rubber septum and a previously published medium [23]. The detailed medium composition is listed in the Supplementary Material (Table S4). The medium was anaerobized through boiling and subsequent gassing with N_2_. Cysteine hydrochloride was added prior to inoculation from a previously anaerobized and sterilized stock solution. Precultures were incubated at a temperature of 37 °C and an agitation rate of 100 RPM (WiseCube WIS-20, Witeg Labortechnik GmbH, Wertheim, Germany). *C. autoethanogenum* precultures were prepared with 5 g L^−1^ xylose as a carbon source. Precultures of *C. ljungdahlii* and *C. ragsdalei* were prepared autotrophically with 1.2 bar CO, 0.4 bar CO_2_, and 0.4 bar H_2_.

### 2.2. Batch Processes in Stirred-Tank Bioreactors

Continuously gassed batch processes were performed in stirred-tank bioreactors with two six-blade Rushton turbines, temperature and pH control, and a working volume of 1 L (*C. autoethanogenum*; KLF2000, Bioengineering AG, Wald, Switzerland; *C. ljungdahlii* and *C. ragsdalei*: Labfors 2, Infors HT, Bottmingen, Switzerland). The reactors were sterilized with 1 L of demineralized water. The medium was autoclaved separately in closed 1 L flasks with butyl rubber septa and transferred through a sterilized silicone tube to the reactor. The medium was anaerobized with a gas mixture of either 60% CO, 20% CO_2_, and 20% H_2_ (*C. autoethanogenum* and *C. ragsdalei*) or 20% CO, 20% CO_2_, 20% H_2_, and 40% N_2_ (*C. ljungdahlii*) with 5 NL h^−1^ (under standard conditions by ISO 10,780 [24]) for at least 12 h at 37 °C. Stirrer speeds were 800 min^−1^ (*C. ljungdahlii* and *C. ragsdalei*) and 1200 min^−1^ (*C. autoethanogenum*), corresponding to a volumetric power input of 3.5 W L^−1^ and 15.1 W L^−1^, respectively. Inoculation was performed using resuspended cells in an anaerobic phosphate saline buffer to achieve an initial optical density OD_600_ of 0.1 in the stirred-tank bioreactor. Batch processes were performed at a temperature of 37 °C, and the pH level was kept constant at pH 6 with the addition of 3 M NaOH or 2 M H_2_SO_4_. Mass flow controllers (*C. autoethanogenum*: Bronkhorst F-101D-RAD-33-V, Wagner Mess- und Regeltechnik, Offenbach, Germany; *C. ljungdahlii* and *C. ragsdalei*: WMR 4000, Brooks Instrument GmbH, Dresden, Germany) ensured a constant total gas flow rate of 5 NL h^−1^ (ISO 10,780 [24]) at either 200 mbar CO_2_, 200 mbar H_2_, and 600 mbar CO (*C. autoethanogenum* and *C. ragsdalei*) or 200 mbar CO_2_, 200 mbar H_2_, 200 mbar CO, and 400 mbar N_2_ (*C. ljungdahlii*) at a total pressure of 1 bar.

### 2.3. Supply of Defined Syngas Impurities

The effect of syngas impurities was studied via the addition of the desired component to the reaction medium prior to inoculation according to previously reported methods [14]. Ammonium was added as NH_4_Cl (1 g L^−1^, 3 g L^−1^, and 6 g L^−1^, respectively). Nitrate was added as NaNO_3_ (0.1 g L^−1^, 0.2 g L^−1^, and 0.5 g L^−1^, respectively). Hydrogen sulfide was added as thioacetamide (TAA) (0.1 g L^−1^, 0.2 g L^−1^, and 0.5 g L^−1^, respectively).

### 2.4. Analytical Methods

#### 2.4.1. Liquid Product Analysis

Samples from the processes with *C. autoethanogenum* were collected through a sampling valve at the bottom of the bioreactor and analyzed by HPLC (LC-2030C Plus, Shimadzu, Kyoto, Japan). Samples from the processes with *C. ljungdahlii* and *C. ragsdalei* were taken using a syringe through a diaphragm at the top of the reactor and analyzed by HPLC (Finnigan Surveyor Plus, Thermo Scientific, Waltham, MA, USA). All samples were analyzed for the main products acetate, ethanol, and 2,3-butanediol, as well as the sugars and metabolites xylose, fructose, and formate. Both HPLC instruments were equipped with a cation exchange column (HPX-87H, Bio-Rad, Munich, Germany) at a column temperature of 60 °C. The elution conditions were isocratic, with 5 mM H_2_SO_4_ as the mobile phase and a constant flow rate of 0.6 mL min^−1^ (*C. autoethanogenum*) and 0.5 mL min^−1^ (*C. ljungdahlii* and *C. ragsdalei*). Both instruments used a refractive index detector (RID) and were combined with a standard series of defined concentrations between 0.05 g L^−1^ and 5.00 g L^−1^ of every measured substance with each measurement. The optical density OD_600_ of the samples was measured using a spectrophotometer (Genesys 10S UV–Vis, Thermo Scientific, Neuss, Germany) at 600 nm, and previously identified individual correlation factors were used to estimate the cell dry weight concentrations (*C. autoethanogenum*: 0.38 ± 0.02 g L^−1^; *C. ljungdahlii*: 0.41 ± 0.01 g L^−1^; *C. ragsdalei*: 0.42 ± 0.03 g L^−1^). Samples for optical density were measured in triplicate for optical densities higher than 0.3.

#### 2.4.2. Online Exhaust Gas Analysis

The exhaust gas was cooled to 2 °C with a reflux condenser prior to analysis. The gas flow rate was measured online by a mass flow meter (Wagner Mess- und Regeltechnik GmbH, Offenbach, Germany), and micro gas chromatography (micro GC 490, Agilent Technologies, Waldbronn, Germany) was applied to measure the concentrations of CO, CO_2_, and H_2_ online in the exhaust gas. The µGC was equipped with three channels with individual separation columns (channel 1: molecular sieve, carrier gas argon, 80 °C, 250 kPa, for the separation of H_2_, N_2_, and CO; channel 2: PlotPQ, carrier gas helium, 80 °C, 150 kPa, for the separation of CO_2_, NH_3_, and NO_x_; channel 3: CP-Sil 5, carrier gas helium, 45 °C, 100 kPa, for the separation of CO_2_, and H_2_S). Every channel uses an individual thermal conductivity detector (TCD). The online data were used to calculate the volumetric uptake or production rates of CO, CO_2_, and H_2_. The individual flow rate of each gas component in the exhaust gas was calculated by multiplying of the individual gas partial pressure of each component measured online by µGC with the total exhaust gas flowrate measured online with mass flow meters. The total consumption of each component was estimated by numerical integration of the individual gas flow rate with a step time of 10 min.

## 3. Results and Discussion

### 3.1. Autotrophic Reference Batch Processes in Continuously Gassed Stirred-Tank Bioreactors

**Figure 1:**
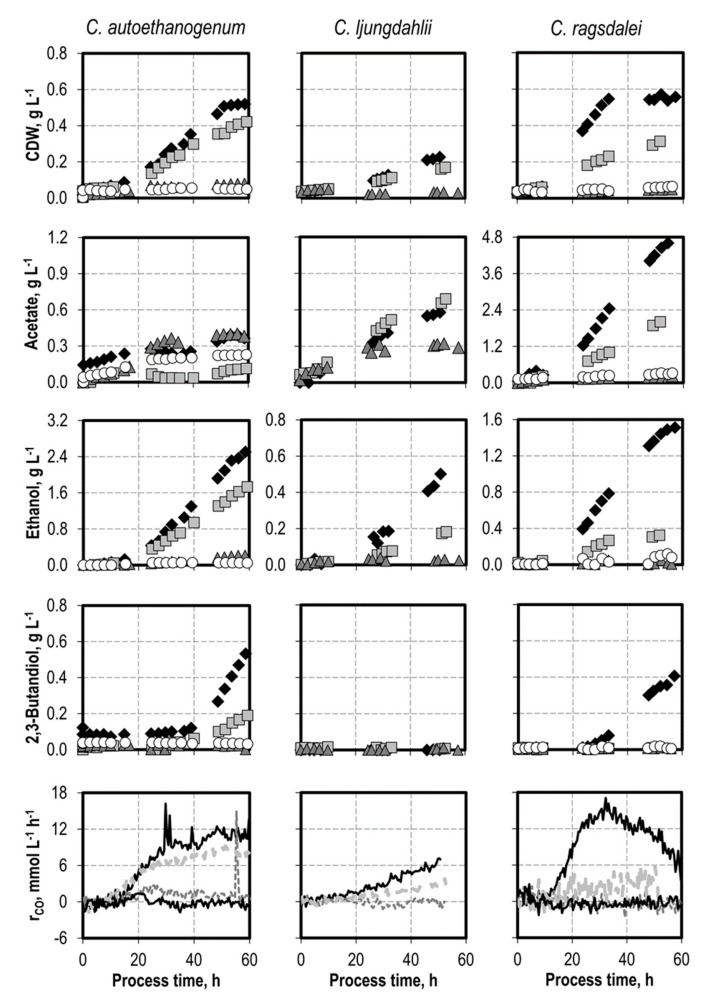
Autotrophic batch processes with *C. autoethanogenum*, *C. ljungdahlii,* and *C. ragsdalei* at varying ammonium chloride concentrations: *C. autoethanogenum*, reference batch process (black diamond, black top line), +1.0 g L^−1^ NH_4_Cl (light grey square, light grey line), +3.0 g L^−1^ NH_4_Cl (dark grey triangle, dark grey line), +5.0 g L^−1^ NH_4_Cl (white circle, black bottom line); *C. ljungdahlii* and *C. ragsdalei*, reference batch process (black diamond, black top line), +3.0 g L^−1^ NH_4_Cl (light grey square, light grey line), +6.0 g L^−1^ NH_4_Cl (dark grey triangle, dark grey line), +9.0 g L^−1^ NH_4_Cl (white circle, black bottom line). The processes were performed in a stirred-tank reactor with continuous gassing (*C. autoethanogenum* and *C. ragsdalei*: 600 mbar CO, 200 mbar CO_2_, and 200 mbar H_2_; *C. ljungdahlii*: 400 mbar N_2_, 200 mbar CO, 200 mbar H_2_, and 200 mbar CO_2_), pH 6 controlled with 3 M NaOH and 2 M H_2_SO_4_, 37 °C, volumetric power input of 15.1 W L^−1^ (*C. autoethanogenum*) and 3.5 W L^−1^ (*C. ljungdahlii* and *C. ragsdalei*). r_CO_ represents the CO uptake rate.

All of the strains were first cultivated without the addition of any syngas impurities as autotrophic reference batch processes (Figure 1). It has to be noted that for the microorganism *C. ljungdahlii*, a reduced CO partial pressure of 200 mbar was chosen as opposed to 600 mbar for *C. autoethanogenum* and *C. ragsdalei*. This reduction was chosen because preliminary studies showed increased growth and ethanol formation for this strain at reduced CO partial pressure (data not shown), and this study aimed to compare the three strains at their best performance. The highest CDW concentrations were observed with *C. ragsdalei* (0.56 g L^−1^), followed by *C. autoethanogenum* (0.52 g L^−1^) and *C. ljungdahlii* (0.23 g L^−1^). *C. ragsdalei* produced considerably more acetate, with a final concentration of 4.61 g L^−1^, whereas *C. autoethanogenum* produced the highest ethanol and 2,3-butanediol concentrations (2.51 g L^−1^ ethanol and 0.53 g L^−1^ 2,3-butanediol, respectively). No 2,3-butanediol formation was observed with *C. ljungdahlii*. CO was the only gaseous substrate to be consumed by all strains. No H_2_ uptake was observed in any of the batch processes. Part of the CO was converted to CO_2_ for providing the necessary reducing equivalents. Further details on total CO consumption and CO_2_ production are given in Tables S1–S3 in the Supplementary Materials. The maximal CO uptake rates varied considerably at ≈16 mmol L^−1^ h^−1^ (*C. ragsdalei*), ≈12 mmol L^−1^ h^−1^ (*C. autoethanogenum*), and ≈6 mmol L^−1^ h^−1^ (*C. ljungdahlii*). Compared to the high volumetric flow rate of the syngas, the CO uptake rates are relatively low, resulting in maximum CO conversions of 11.9% (*C. ragsdalei*), 9.0% (*C. autoethanogenum*), and 13.4% (*C. ljungdahlii*), which does not support any kind of limitation by the carbon input.

The final alcohol to acetate ratio achieved in the autotrophic reference batch processes exhibited a distinct maximum using *C. autoethanogenum* (7.60 g g^−1^), as compared to 0.86 g g^−1^ with *C. ljungdahlii* or 0.41 g g^−1^ with *C. ragsdalei.* The high CO uptake rates observed with *C. ragsdalei* mainly resulted in high acetate production. High organic acid production rates and concentrations have been associated with a failure to trigger alcohol production [25,26], as well as with higher ATP maintenance costs [27].

The observed maximum specific growth rate (0.050 h^−1^) and maximum CO uptake rate (6 mmol L^−1^ h^−1^) of *C. ljungdahlii* were in accordance with the published data [28]. The reported maximum specific growth rates of *C. ragsdalei* varied considerably, e.g., 0.175 h^−1^ [8] or 0.065 h ^−1^ [29]. The growth rate observed in this study was in line with the reported data (0.116 h^−1^). The measured maximum specific growth rate of *C. autoethanogenum* (0.065 h^−1^) exceeded that of the data from the literature, e.g., 0.042 h^−1^ achieved with a gas mixture of 2% CO, 23% CO_2_, and 65% H_2_ [30].

### 3.2. Defined Addition of Impurities: Ammonium

Ammonium was supplemented as NH_4_Cl before inoculation with *C. autoethanogenum* (+ 1.0 g L^−1^, + 3.0 g L^−1^, and + 5.0 g L^−1^ NH_4_Cl), *C. ljungdahlii* (+ 3.0 g L^−1^, and + 6.0 g L^−1^ NH_4_Cl), and *C. ragsdalei* (+ 3.0 g L^−1^, + 6.0 g L^−1^, and + 9.0 g L^−1^ NH_4_Cl) (see Figure 1). The initial addition of 3.0 g L^−1^ NH_4_Cl reduced the final CDW concentration of *C. autoethanogenum* by 85%, of *C. ljungdahlii* by 26%, and of *C. ragsdalei* by 45%. Growth inhibition was observed for *C. autoethanogenum* after the addition of 5.0 g L^−1^ NH_4_Cl and for *C. ljungdahlii* and *C. ragsdalei* after the addition of 6.0 g L^−1^ NH_4_Cl. The inhibitory effect of NH_4_Cl addition was also reflected in the observed decrease in the CO uptake rates in all batch processes after supplementation with ammonium. Alcohol production was reduced after any initial addition of NH_4_Cl. The supplementation with 3.0 g L^−1^ NH_4_Cl prevented any formation of 2,3-butanediol with *C. autoethanogenum* and *C. ragsdalei*.

### 3.3. Defined Addition of Impurities: Nitrate

**Figure 2:**
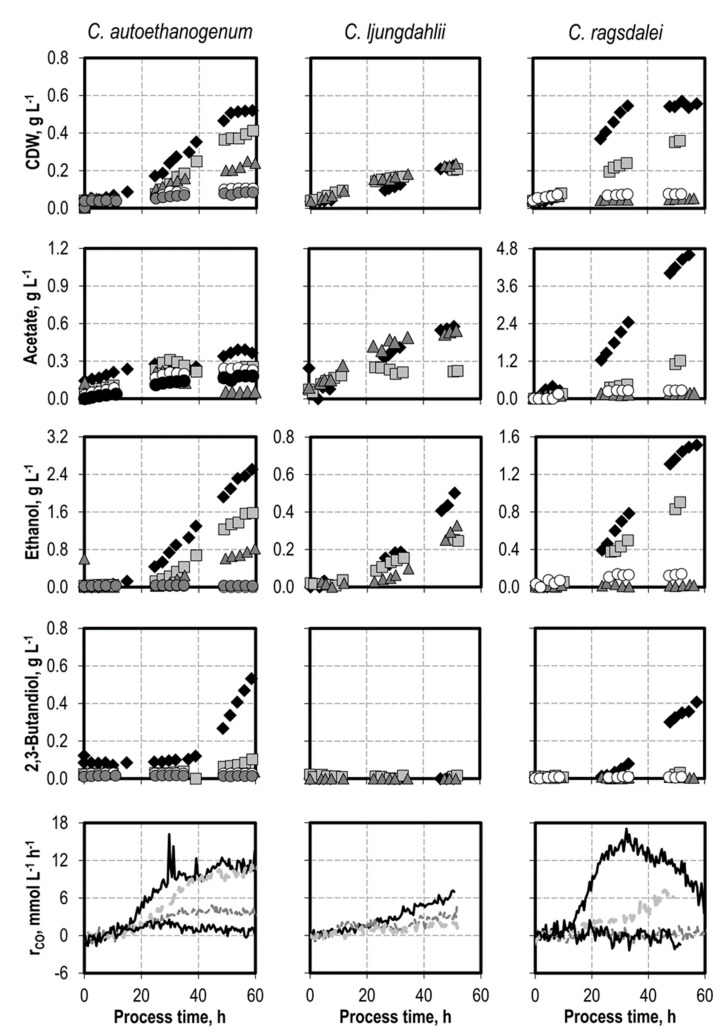
Autotrophic batch processes with *C. autoethanogenum*, *C. ljungdahlii*, and *C. ragsdalei* at varying NaNO_3_ concentrations: *C. autoethanogenum*, reference batch process (black diamond, black top line), +0.1 g L^−1^ NaNO_3_ (light grey square, light grey line), +0.2 g L^−1^ NaNO_3_ (dark grey triangle, dark grey line), +0.5 g L^−1^ NaNO_3_ (white dircle, black bottom line), +1.0 g L^−1^ NaNO_3_ (dark grey circle); *C. ljungdahlii* and *C. ragsdalei*, reference batch process (black diamond, black top line), +0.1 g L^−1^ NH_4_Cl (light grey square, light grey line), +0.5 g L^−1^ NaNO_3_ (dark grey triangle, dark grey line), +1.0 g L^−1^ NaNO_3_ (white circle, black bottom line). The batch processes were performed in a stirred-tank reactor with continuous gassing (*C. autoethanogenum* and *C. ragsdalei*: 600 mbar CO, 200 mbar CO_2_, and 200 mbar H_2_; *C. ljungdahlii*: 400 mbar N_2_, 200 mbar CO, 200 mbar H_2_, and 200 mbar CO_2_), pH 6 controlled with 3 M NaOH and 2 M H_2_SO_4_, 37 °C, volumetric power input of 15.1 W L^−1^ (*C. autoethanogenum*) and 3.5 W L^−1^ (*C. ljungdahlii* and *C. ragsdalei*). r_CO_ represents the CO uptake rate.

Nitrate was added as NaNO_3_ before inoculation with *C. autoethanogenum* (0.1 g L^−1^, 0.2 g L^−1^, 0.5 g L^−1^, and 1.0 g L^−1^ NaNO_3_), *C. ljungdahlii* (0.1 g L^−1^, and 0.5 g L^−1^ NaNO_3_), and *C. ragsdalei* (0.1 g L^−1^, 0.2 g L^−1^, and 0.5 g L^−1^ NaNO_3_) (see Figure 2). Adding nitrate slowed the growth of *C. autoethanogenum* and *C. ragsdalei* in all of the nitrate concentrations studied. The complete growth inhibition of *C. ragsdalei* was observed after adding 0.5 g L^−1^ NaNO_3_. *C. ljungdahlii* exhibited the highest tolerance after nitrate additions, with little difference in the final cell dry weight concentrations in all cases, but a clearly shorter exponential growth phase.

No net production of acetate was observed with *C. ljungdahlii* in the final 30 h of the batch processes with 0.1 g L^−1^ NaNO_3_ and 0.5 g L^−1^ NaNO_3_, respectively. A strong reduction of acetate production occurred in the batch processes with *C. ragsdalei,* which was independent of the initial NaNO_3_ concentration. Nitrate is known to increase the ATP/ADP ratio in *C. ljungdahlii* [21]. More ATP can thus be provided through nitrate reduction with less acetate production for biomass formation.

Ethanol production was reduced in all of the batch processes with the addition of NaNO_3_. *C. ljungdahlii* showed the lowest reduction of final ethanol concentrations. 2,3-Butanediol formation was strongly inhibited by nitrate, and no 2,3-butanediol was detected with *C. ljungdahlii* and *C. ragsdalei*. A reduced formation of 0.10 g L^−1^ 2,3-butanediol was observed with *C. autoethanogenum* at 0.1 g L^−1^ NaNO_3_.

### 3.4. Defined Addition of Impurities: Hydrogen Sulfide

**Figure 3:**
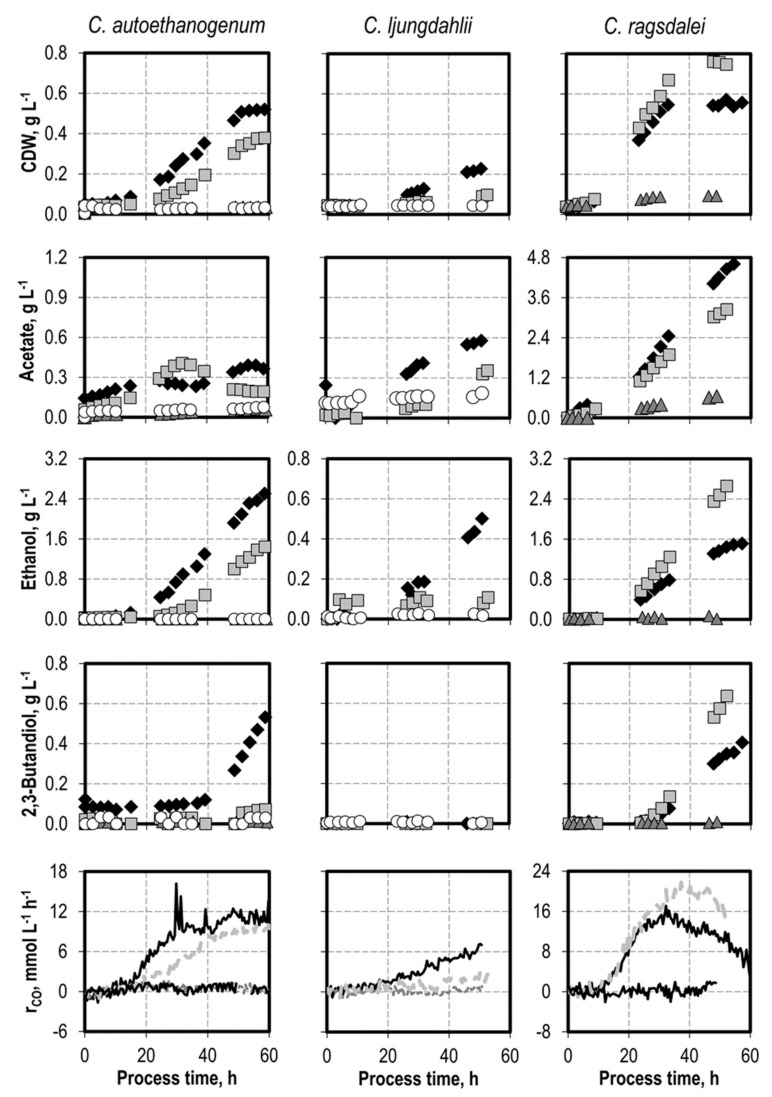
Autotrophic batch processes with *C. autoethanogenum*, *C. ljungdahlii*, and *C. ragsdalei* at varying initial H_2_S concentrations supplied by addition of thioacetamide: *C. autoethanogenum*, reference batch process (black diamond, black top line), +0.1 g L^−1^ H_2_S (light grey square, light grey line), +0.3 g L^−1^ H_2_S (dark grey triangle, dark grey line), +0.5 g L^−1^ H_2_S (white circle, black bottom line); *C. ljungdahlii* and *C. ragsdalei*, reference batch process (black diamond, black top line), +0.1 g L^−1^ H_2_S (light grey square, light grey line), +0.2 g L^−1^ NH_4_Cl (dark grey triangle, dark grey line), +0.5 g L^−1^ NH_4_Cl (white circle, black bottom line). The batch processes were performed in a stirred-tank reactor with continuous gassing (*C. autoethanogenum* and *C. ragsdalei*: 600 mbar CO, 200 mbar CO_2_, and 200 mbar H_2_; *C. ljungdahlii*: 400 mbar N_2_, 200 mbar CO, 200 mbar H_2_, and 200 mbar CO_2_), pH 6 controlled with 3 M NaOH and 2 M H_2_SO_4_, 37 °C, volumetric power input of 15.1 W L^−1^ (*C. autoethanogenum*) and 3.5 W L^−1^ (*C. ljungdahlii* and *C. ragsdalei*). r_CO_ represents the CO uptake rate.

Hydrogen sulfide was added as thioacetamide (TAA) before inoculation with *C. autoethanogenum* (0.1 g L^−1^ H_2_S, 0.3 g L^−1^, and 0.5 g L^−1^), as well as with *C. ljungdahlii* and *C. ragsdalei* (0.1 g L^−1^, and 0.5 g L^−1^ H_2_S) (see Figure 3).

All of the TAA concentrations investigated decreased the cell dry weight concentrations; the final product concentrations; and, correspondingly, the CO uptake rates of *C. autoethanogenum* and *C. ljungdahlii*. Adding 0.3 g L^−1^ H_2_S completely inhibited the growth and CO uptake of *C. autoethanogenum*. No growth of *C. ljungdahlii* and *C. ragsdalei* was observed with 0.5 g L^−1^ H_2_S. A concentration of 0.1 g L^−1^ H_2_S induced a lag phase of 20 h with *C. autoethanogenum*, but it increased acetate production in the first 30 h. The acetate concentration later decreased until the end of the process. 2,3-Butanediol formation of *C. autoethanogenum* was strongly inhibited in the batch process with 0.1 g L^−1^ H_2_S. The addition of 0.1 g L^−1^ H_2_S resulted in less biomass formation with *C. ljungdahlii*, a delayed acetate production, and no alcohol formation.

The addition of 0.1 g L^−1^ H_2_S increased the final cell dry weight concentration of *C. ragsdalei* by 34%. Higher CO uptake rates were also observed, with maximal CO uptake rates reaching approximately 21 mmol L^−1^ h^−1^. Product formation shifted from acetate to alcohol production, with not only higher final concentrations of ethanol and 2,3-butanediol when compared with the reference batch process for each strain, but also higher biomass-related yields of both alcohols. This effect might occur due to the additional presence of a sulfur source or to the reducing effect of H_2_S, which may lead to a more reduced redox potential in the cultivation medium and, thus, an increase in growth and reduction of acetate to ethanol.

### 3.5. Comparison of Clostridial Strains

**Figure 4:**
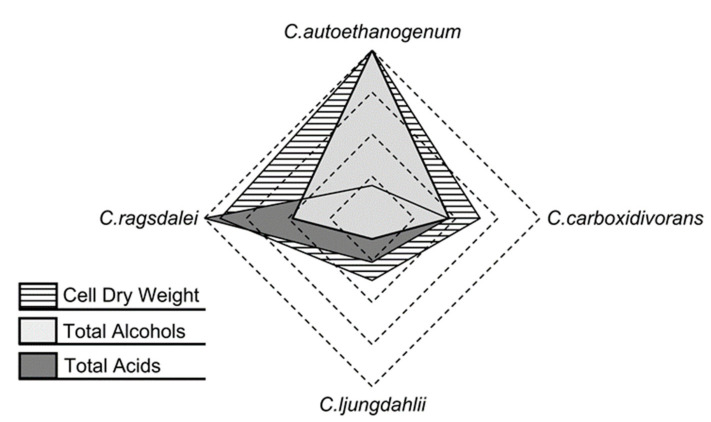
Comparison of the autotrophic reference batch processes with *C. autoethanogenum*, *C. ljungdahlii*, *C. ragsdalei*, and *C. carboxidivorans* (data for *C. carboxidivorans* extracted from previously published results in [14]) with respect to maximum CDW concentrations, maximum total concentrations of alcohols on a C mol basis, and maximum total concentration of acids on a C mol basis.

The results with the acetogens *C. autoethanogenum*, *C. ljungdahlii*, and *C. ragsdalei* of this study were compared to previously published reference data with *C. carboxidivorans* [14], which have been measured in batch operated stirred-tank bioreactors at comparable reaction conditions. An overall comparison of the autotrophic reference batch process performances of the three strains studied, including the published results for *C. carboxidivorans* [14], shows that *C. autoethanogenum* was able to produce the highest amounts of biomass and alcohols while also maintaining low acid concentrations (Figure 4). *C. ragsdalei* achieved 91% (*w*/*w*) of the maximum cell dry weight concentration measured with *C. autoethanogenum* and further produced the highest amounts of organic acids. *C. ljungdahlii* showed the lowest production of biomass, organic acids, and alcohols.

**Figure 5:**
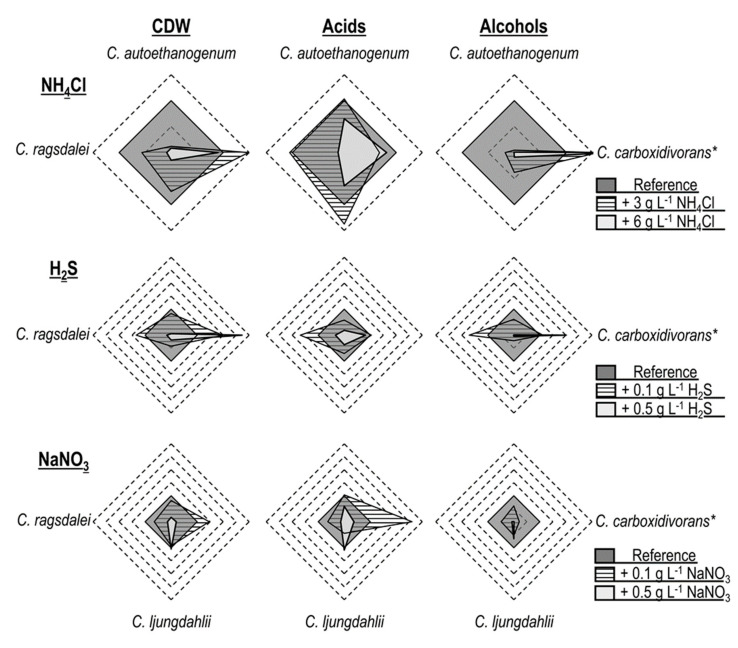
Comparison of autotrophic batch processes with *C. autoethanogenum*, *C. ljungdahlii*, *C. ragsdalei*, and C. carboxidivorans*. All values in the radar plots were calculated relative to the individual maximum concentration observed with the indicated microorganism (reference process = 1.0). * Data of C. carboxidivorans extracted from previously published results in [14], with concentrations of +5.0 g L^−1^ NH_4_Cl, +0.1 g L^−1^ H_2_S, and +0.1 g L^−1^ NaNO_3_ 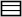, or +7.5 g L^−1^ NH_4_Cl, +0.5 g L^−1^ H_2_S, and +1.0 g L^−1^ NaNO_3_ 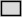.

The addition of ammonium favored biomass and alcohol formation in the autotrophic batch processes with *C. carboxidivorans* [14] while reducing the productivity of the other three *Clostridial* strains (Figure 5). *C. autoethanogenum* demonstrated the lowest tolerance to NH_4_Cl, with growth inhibition already occurring with the supplementation of 3.0 g L^−1^ NH_4_Cl, whereas *C. ljungdahlii* and *C. ragsdalei* showed inhibition with the supplementation of 6.0 g L^−1^ NH_4_Cl.

H_2_S addition promoted biomass, acid, and alcohol formation in the autotrophic batch processes with *C. ragsdalei* at a low initial concentration (0.1 g L^−1^ H_2_S). *C. carboxidivorans* biomass production was increased with both added sulfide concentrations, whereby an increase in the formation of alcohols was solely observed with 0.5 g L^−1^ H_2_S [14]. The other strains exhibited reduced productivities after H_2_S addition, with *C. ljungdahlii* showing a strong inhibition to sulfide. The addition of 0.5 g L^−1^ H_2_S resulted in strong inhibitions of *C. autoethanogenum*, *C. ljungdahlii*, and *C. ragsdalei* and thus represents a critical impurity in the syngas fermentation with these strains.

Nitrate slowed or inhibited the biomass formation of *C. autoethanogenum* and *C. ragsdalei*, with the effect increasing at higher initial nitrate concentrations. *C. ljungdahlii* was, in turn, less influenced by nitrate, leading to equivalent cell dry weight concentrations with and without nitrate, as well as showing a higher tolerance to this impurity. The alcohol formation in all of the strains studied was suppressed by nitrate, including *C. carboxidivorans* [14], but *C. carboxidivorans* was stimulated by the addition of 0.1 g L^−1^ NaNO_3_ and produced more biomass and organic acids [14].

Overall, the three *Clostridial* strains studied exhibited similar responses to the added impurities. These responses were, in turn, very different from those published with *C. carboxidivorans* [14], which showed more robustness to the effects of the syngas impurities NH_3_ and H_2_S. Since *C. carboxidivorans* is genetically not as closely related to the other three studied strains [4,5], it is not surprising that its response to the defined impurities should differ. *C. autoethanogenum, C. ljungdahlii*, and *C. ragsdalei* were generally inhibited by the addition of H_2_S (as TAA), nitrate, and ammonium, with the exception of the addition of 0.1 g L^−1^ H_2_S to the autotrophic batch process with *C. ragsdalei*. As a consequence, the accumulation of 0.1 g L^−1^ H_2_S, 0.073 g L^−1^ NO_3_^−1^ (equivalent to 0.1 g L^−1^ NaNO_3_), and 2.14 g L^−1^ NH_4_^+^ (equivalent to 6.3 g L^−1^ NH_4_Cl) in fermentation processes with real syngas should be avoided.

Further details on total CO consumption, total CO_2_ production, carbon balance recoveries, specific growth rates, and the maximum concentrations of CDW and products are provided in the Supplementary Materials (Tables S1–S3) for all the batch processes described in this work using the individual microorganisms (*C. autoethanogenum*, *C. ljungdahlii*, and *C. ragsdalei*, respectively).

The identified inhibiting concentrations of impurities in the liquid phase were used to estimate the corresponding concentrations in the gas phase and, therefore, provide a quantitative goal respecting the quality requirements of real syngases. The typical orders of magnitude for trace impurities in real biogenic syngas from entrained flow gasification of biogenic residues are 4500 ppm NH_3_, 500 ppm H_2_S, and 200 ppm NO_x_ [31,32]. The corresponding concentrations in a syngas were estimated given the assumption of complete absorption of the syngas trace component in the liquid phase within a process time of 60 h, as previously described [14]. Typical solubilities of the investigated gas impurities in pure water at 25 °C and 1013.25 mbar partial pressure are 0.1876 mol NH_3_ mol^−1^ H_2_O, 1.830∙10^−3^ mol H_2_S mol^−1^ H_2_O, 3.477∙10^−5^ mol NO mol^−1^ H_2_O, and 1.488∙10^−4^ mol NO_2_ mol^−1^ H_2_O [33]. These concentrations correspond to 199.7 g L^−1^ NH_3_, 3.90 g L^−1^ H_2_S, 0.065 g L^−1^ NO, and 1.28 g L^−1^ NO_2_, respectively. However, it has to be noted that these concentrations do not take any chemical reaction into account [33]. All investigated concentrations of NH_4_^+^, H_2_S, and NO_3_^−^ are in the range of these typical solubilities. It should be noted that the impurities threshold identified in this study represents a conservative limit, since in a continuously gassed process, the impurities would gradually accumulate in the cell broth. Thus, the growth phase would occur at lower impurity concentrations than the one in this study, leading presumably to higher CDW and product concentrations and the adaption of the cells.

The initial addition of 3.0 g L^−1^ NH_4_Cl in the liquid phase was found to be inhibiting for growth and product formation of *C. autoethanogenum*, *C. ljungdahlii*, and *C. ragsdalei* in the autotrophic batch processes. Under the given assumptions, 3.0 g L^−1^ NH_4_Cl would be reached at a concentration of 4560 ppm NH_3_ in the gas phase within a total batch process time of 60 h. Since the standard medium in all of the autotrophic batch processes already contained 3.3 g L^−1^ NH_4_Cl [23], a reduction of the initially supplied NH_4_Cl could reduce the inhibiting effect of NH_3_ provided by a typical biogenic syngas (4500 ppm NH_3_). It has already been shown that a reduction of the initial ammonium concentration in the medium by 50% did not influence biomass growth or product formation of *C. ragsdalei* [18]. Additionally, with the continuous gassing, the ammonium concentration would increase with process time rather than remaining constant, enabling a possible adaption of the cells. The growth of *C. ragsdalei* resumed with 6.0 g L^−1^ and 9.0 g L^−1^ NH_4_Cl after 100 h (data not shown), and growth of *C. autoethanogenum* resumed with 3.0 g L^−1^ NH_4_Cl after 78 h (data not shown), indicating that an adaption to increasing ammonium concentrations may be possible.

The initial addition of 0.1 g L^−1^ NaNO_3_ was found to inhibit growth and alcohol production with *C. ragsdalei*, and *C. autoethanogenum*. An amount of 0.1 g L^−1^ NaNO_3_ would correspond to a gas phase concentration of 118 ppm NO_x_ after a process time of 60 h if the entire amount of nitrogen from the NO_x_ was absorbed in the liquid phase and converted into NO_3_^−^. Therefore, the purification of a typical biogenic syngas at 200 ppm NO_x_ would be necessary to ensure a stable process without reduced alcohol production. However, the growth of *C. ljungdahlii* was not affected, and its alcohol production was only slightly reduced by the addition of NaNO_3_ concentrations of up to 0.5 g L^−1^ NaNO_3_. Under the same assumptions, a concentration of 0.5 g L^−1^ NaNO_3_ would be reached after 60 h with a syngas at 588 ppm NO_x_. Thus, a biogenic syngas at 200 ppm NO_x_ might not need a further purification for this trace component if *C. ljungdahlii* were applied for syngas fermentation.

An initial concentration of 0.1 g L^−1^ H_2_S was found to inhibit growth as well as alcohol production of *C. autoethanogenum* and *C. ljungdahlii*. An amount of 0.1 g L^−1^ H_2_S corresponds to 108 ppm H_2_S in the syngas within a batch process time of 60 h. The inhibiting concentration for *C. ragsdalei* of 0.5 g L^−1^ H_2_S corresponds to a concentration of 540 ppm H_2_S in the gas phase for 60 h. Thus, a biogenic syngas at a typical concentration of 200 ppm H_2_S would not be critical in autotrophic batch processes with *C. ragsdalei*. If *C. autoethanogenum* or *C. ljungdahlii* were applied for syngas fermentation, H_2_S separation from the biogenic syngas would be necessary.

## 4. Conclusions

*C. autoethanogenum* was shown to produce the highest cell dry weight and alcohol concentrations in continuously gassed batch processes without the addition of syngas impurities, as compared to *C. ljungdahlii* and *C. ragsdalei*. Syngas impurities such as NH_3_, NO_x_, and H_2_S critically impacted batch processes with *C. autoethanogenum*, *C. ljungdahlii*, and *C. ragsdalei* to varying extents, thus differing from published results with *C. carboxidivorans* [14]. The results presented herein offer an initial setpoint for the quality requirements of real syngas with respect to the impurities tested. Further investigation of the possible combinatory effects of these impurities are of utmost relevance.

## Data Availability

The original contributions presented in the study are included in the article/Supplementary Materials; further inquiries can be directed to the corresponding authors.

## Supplementary Materials

The following supporting information can be downloaded at https://www.mdpi.com/article/10.3390/microorganisms10040681/s1, Table S1: Total consumption of CO and production of CO_2_, biomass, and products on a C-mol basis and carbon balances, as well as maximum CDW concentrations, maximum product concentrations, and the maximum specific growth rate of the autotrophic batch processes with *C. autoethanogenum*, *C. ljungdahlii*, and *C. ragsdalei* in continuously gassed stirred-tank bioreactors (V = 1 L) with the initial addition of NH_4_Cl, Table S2: Total consumption of CO and production of CO_2_, biomass, and products on a C-mol basis and carbon balances, as well as maximum CDW concentrations, maximum product concentrations, and the maximum specific growth rate of the autotrophic batch processes with *C. autoethanogenum*, *C. ljungdahlii*, and *C. ragsdalei* in continuously gassed stirred-tank bioreactors (V = 1 L) with the initial addition of H_2_S, Table S3: Total consumption of CO and production of CO_2_, biomass, and products on a C-mol basis and carbon balances, as well as maximum CDW concentrations, maximum product concentrations, and the maximum specific growth rate of the autotrophic batch processes with *C. autoethanogenum*, *C. ljungdahlii*, and *C. ragsdalei* in continuously gassed stirred-tank bioreactors (V = 1 L) with the initial addition of NaNO_3_, Table S4: Composition of the liquid medium previously described by Doll et al. [23] for precultures in anaerobic shaken bottles and batch processes in stirred-tank bioreactors.
