# Supplementary material for: Comparison of Syngas-Fermenting Clostridia in Stirred-Tank Bioreactors and the Effects of Varying Syngas Impurities
Source: Microorganisms. 2022 Mar 22;10(4):681. doi: 10.3390/microorganisms10040681 (PMC9032146; doi:10.3390/microorganisms10040681)
Supplement: Supplementary file 1 [file microorganisms-10-00681-s001.zip › microorganisms-1634193-supplementary.pdf]

microorganisms

MDPI

Supplementary Material

Table S1. Total consumption of CO and production of CO_2, biomass, and products on a C-mol basis and carbon balances, as well as maximum CDW concentrations, maximum product concentrations, and the maximum specific growth rate of the autotrophic batch processes with *C. autoethanogenum*, *C. ljungdahlii*, and *C. ragsdalei* in continuously gassed stirred-tank bioreactors (V = 1 L) with the initial addition of NH_4Cl.

	Reference process	+ 1.0 g L^{-1} NH_4Cl	+ 3.0 g L^{-1} NH_4Cl	+ 5.0 g L^{-1} NH_4Cl	
C. autoethanogenum					
Carbon in medium, mmol C	9.94	9.94	9.94	9.94	
CO consumption, mmol C	377.32	263.56	<50	<50	
CO_2 production, mmol C	244.03	164.08	<20	<20	
$CO:CO_2$ ratio, -	1.55	1.61	-	-	
Carbon in products, mmol C	126.44	81.11	22.25	10.20	
Carbon in biomass, mmol C	16.58	13.36	2.55	1.56	
C-balance (recovery),%	99.95%	94.53%	-	-	
CDW_{max}, g L^{-1}	0.52	0.42	0.08	0.0551	
$c_{Acetate, max}$, g L^{-1}	0.39	0.11	0.40	0.23	
$c_{Ethanol, max}$, g L^{-1}	2.53	1.73	0.22	0.06	
$c_{2,3\text{-}Butandiol, max}$, g L^{-1}	0.57	0.19	0.03	0.04	
μ_{max}, h^{-1}	0.065	0.057	0.016	0.003	
C. ljungdahlii				+ 6.0 g L^{-1} NH_4Cl	+ 9.0 g L^{-1} NH_4Cl
Carbon in medium, mmol C	9.94		9.94	9.94	
CO consumption, mmol C	115.92		64.13	-	
CO_2 production, mmol C	49.71		39.23	-	
$CO:CO_2$ ratio, -	2.33		1.63	-	
Carbon in products, mmol C	49.65		34.51	11.65	
Carbon in biomass, mmol C	7.80		5.42	-0.02	
C-balance (recovery),%	85.14%		106.87%	-	
CDW_{max}, g L^{-1}	0.227		0.170	0.033	
$c_{Acetate, max}$, g L^{-1}	0.578		0.690	0.321	
$c_{Ethanol, max}$, g L^{-1}	0.501		0.183	0.030	
$c_{2,3\text{-}Butandiol, max}$, g L^{-1}	0.000		0.008	0.013	
μ_{max}, h^{-1}	0.050		0.033	0.012	
C. ragsdalei					
Carbon in medium, mmol C	9.94		9.94	9.94	9.94
CO consumption, mmol C	571.59		174.41	0.03	-
CO_2 production, mmol C	339.14		90.34	1.96	-
$CO:CO_2$ ratio, -	1.69		1.93	-	-
Carbon in products, mmol C	271.08		93.07	2.26	10.76

Carbon in biomass, mmol C	21.75		11.43	0.31	1.09
C-balance (recovery),%	108.67%		105.69%	-	-
CDW_{max}, g L^{-1}	0.557		0.314	0.047	0.066
$c_{Acetate,\ max}$, g L^{-1}	4.935		2.011	0.228	0.320
$c_{Ethanol,\ max}$, g L^{-1}	1.512		0.325	0.005	0.081
$c_{2,3\text{-}Butandiol,\ max}$, g L^{-1}	0.406		0.012	0.005	0.006
μ_{max}, h^{-1}	0.116		0.065	0.055	-

Table S2. Total consumption of CO and production of CO_2, biomass, and products on a C-mol basis and carbon balances, as well as maximum CDW concentrations, maximum product concentrations, and the maximum specific growth rate of the autotrophic batch processes with *C. autoethanogenum, C. ljungdahlii,* and *C. ragsdalei* in continuously gassed stirred-tank bioreactors (V = 1 L) with the initial addition of H_2S.

C. autoethanogenum	Reference process	+ 0.1 g L^{-1} H_2S	+ 0.3 g L^{-1} H_2S	+ 0.5 g L^{-1} H_2S
Carbon in medium, mmol C	9.94	12.60	17.93	23.25
CO consumption, mmol C	377.32	239.41	<50	<50
CO_2 production, mmol C	244.03	162.71	<20	<20
CO:CO_2 ratio, -	1.55	1.47	-	-
Carbon in products, mmol C	126.44	69.95	2.34	4.05
Carbon in biomass, mmol C	16.58	11.99	1.02	1.05
C-balance (recovery),%	99.95%	97.08%	-	-
CDW_{max}, g L^{-1}	0.52	0.38	0.04	0.04
$c_{Acetate,\ max}$, g L^{-1}	0.39	0.41	0.07	0.08
$c_{Ethanol,\ max}$, g L^{-1}	2.53	1.44	0.00	0.00
$c_{2,3\text{-}Butandiol,\ max}$, g L^{-1}	0.57	0.07	0.01	0.03
μ_{max}, h^{-1}	0.065	0.054	0.033	0.002
C. ljungdahlii				
Carbon in medium, mmol C	9.94	12.60		23.25
CO consumption, mmol C	115.92	53.32		1.63
CO_2 production, mmol C	49.71	-9.93		-6.04
CO:CO_2 ratio, -	2.33	-		-
Carbon in products, mmol C	49.65	18.13		2.74
Carbon in biomass, mmol C	7.80	2.13		0.09
C-balance (recovery),%	85.14%	-		-
CDW_{max}, g L^{-1}	0.227	0.097		0.043
$c_{Acetate,\ max}$, g L^{-1}	0.578	0.355		0.186
$c_{Ethanol,\ max}$, g L^{-1}	0.501	0.108		0.017
$c_{2,3\text{-}Butandiol,\ max}$, g L^{-1}	0.000	0.000		0.006
μ_{max}, h^{-1}	0.050	0.020		-
C. ragsdalei				
Carbon in medium, mmol C	9.94	12.60		23.25

CO consumption, mmol C	571.59	630.94		5.38
CO_2 production, mmol C	339.14	337.14		25.68
CO:CO_2 ratio, -	1.69	1.87		-
Carbon in products, mmol C	271.08	284.18		24.85
Carbon in biomass, mmol C	21.75	28.96		2.16
C-balance (recovery),%	108.67%	101.05%		-
CDW_{max}, g L^{-1}	0.557	0.747		0.091
$c_{Acetate, max}$, g L^{-1}	4.935	3.246		0.649
$c_{Ethanol, max}$, g L^{-1}	1.512	2.658		0.011
$c_{2,3\text{-}Butandiol, max}$, g L^{-1}	0.406	0.638		0.010
μ_{max}, h^{-1}	0.116	0.108		0.025

Table S3. Total consumption of CO and production of CO_2, biomass, and products on a C-mol basis and carbon balances, as well as maximum CDW concentrations, maximum product concentrations, and the maximum specific growth rate of the autotrophic batch processes with *C. autoethanogenum, C. ljungdahlii,* and *C. ragsdalei* in continuously gassed stirred-tank bioreactors (V = 1 L) with the initial addition of $NaNO_3$.

C. autoethanogenum	Reference process	+ 0.1 g L^{-1} $NaNO_3$	+ 0.2 g L^{-1} $NaNO_3$	+ 0.5 g L^{-1} $NaNO_3$	+ 1.0 g L^{-1} $NaNO_3$
Carbon in medium, mmol C	9.94	9.94	9.94	9.94	9.94
CO consumption, mmol C	377.32	242.81	89.76	<50	<50
CO_2 production, mmol C	244.03	167.96	54.69	<20	<20
CO:CO_2 ratio, -	1.55	1.45	1.64	-	-
Carbon in products, mmol C	126.44	78.08	37.75	7.69	7.36
Carbon in biomass, mmol C	16.58	13.08	7.60	3.32	2.70
C-balance (recovery),%	99.95%	102.52%	100.34%	-	-
CDW_{max}, g L^{-1}	0.52	0.41	0.25	0.11	0.09
$c_{Acetate, max}$, g L^{-1}	0.39	0.31	0.22	0.25	0.18
$c_{Ethanol, max}$, g L^{-1}	2.53	1.58	0.82	0.03	0.04
$c_{2,3\text{-}Butandiol, max}$, g L^{-1}	0.57	0.10	0.04	0.03	0.02
μ_{max}, h^{-1}	0.065	0.059	0.038	0.028	0.018
C. ljungdahlii					
Carbon in medium, mmol C	9.94	9.94		9.94	
CO consumption, mmol C	115.92	51.06		95.07	
CO_2 production, mmol C	49.71	30.03		57.43	
CO:CO_2 ratio, -	2.33	1.70		1.66	
Carbon in products, mmol C	49.65	21.61		38.61	
Carbon in biomass, mmol C	7.80	6.85		8.09	
C-balance (recovery),%	85.14%	95.89%		99.16%	
CDW_{max}, g L^{-1}	0.227	0.209		0.238	
$c_{Acetate, max}$, g L^{-1}	0.578	0.224		0.547	
$c_{Ethanol, max}$, g L^{-1}	0.501	0.246		0.328	

$c_{2,3\text{-Butandiol, max}}$, g L^{-1}	0.000	0.016	0.000
μ_{max}, h^{-1}	0.050	0.076	0.061
C. ragsdalei			
Carbon in medium, mmol C	9.94	9.94	9.94
CO consumption, mmol C	571.59	213.63	0.24
CO_2 production, mmol C	339.14	116.41	19.54
$CO:CO_2$ ratio, -	1.69	1.84	-
Carbon in products, mmol C	271.08	95.17	14.81
Carbon in biomass, mmol C	21.75	13.04	1.40
C-balance (recovery),%	108.67%	100.47%	-
CDW_{max}, g L^{-1}	0.557	0.360	0.077
$c_{\text{Acetate, max}}$, g L^{-1}	4.935	1.217	0.262
$c_{\text{Ethanol, max}}$, g L^{-1}	1.512	0.904	0.132
$c_{2,3\text{-Butandiol, max}}$, g L^{-1}	0.406	0.031	0.005
μ_{max}, h^{-1}	0.116	0.059	0.013

Table S4. Composition of the liquid medium previously described by Doll *et al.* [23] for precultures in anaerobic shaken bottles and batch processes in stirred-tank bioreactors.

Component	Formula	Concentration in Stock solution, g L^{-1}
**Mineral Solution**		**33.3x**
**ammoniumchloride**	NH_4Cl	100
**natrium chloride**	NaCl	80
**potassium chloride**	KCl	10
**potassium dihydrogen phosphate**	KH_2PO_4	10
**magnesium sulfate**	$MgSO_4$	20
**Calcium chloride**	$CaCl_2$	4
**Vitamin solution**		**100x**
**pyridoxine**	$C_8H_{11}NO_3$	0.01
**thiamine**	$C_{12}H_{17}ClN_4OS$	0.005
**riboflavine**	$C_{17}H_{20}N_4O_6$	0.005
**calcium pantothenate**	$Ca(C_9H_{16}NO_5)_2$	0.005
**liponic acid**	$C_8H_{14}O_2S_2$	0.005
**para amino benzoic acid**	$C_7H_7NO_2$	0.005
**nicotinic acid**	$C_6H_5NO_2$	0.005
**Vitamin B12**	$C_{72}H_{100}CoN_{18}O_{17}P$	0.005
**D-biotine**	$C_{10}H_{16}N_2O_3S$	0.002
**folic acid**	$C_{19}H_{19}N_7O_6$	0.002
**2 mercapto ethane sulfonic acid**	$C_2H_6O_3S_2$	0.02
**Trace element solution**		**100x**
**Nitrilotriacetic acid**	$C_6H_9NO_6$	2.00
**Mangan sulfate**	$MnSO_4$	1.00
**Ammonium iron sulfate**	$NH_4Fe(SO_4)_2$	0.80
**cobalt chloride**	$CoCl_2$	0.20
**zinc sulfate**	$ZnSO_4$	0.20
**copper chloride**	$CuCl_2$	0.02
**nickel chloride**	$NiCl_2$	0.02

**sodium molybdate**	Na_2MoO_4	0.02
**sodium selenate**	Na_2SeO_4	0.02
**sodium wolframate**	Na_2WO_4	0.02
		**medium concentration**
**yeast extract**		1.0 g L^{-1}
**cysteine hydrochloride**	$C_3H_7NO_2S$ HCl	0.4 g L^{-1}
**morpholino ethane sulfonic acid**[a)]	$C_6H_{13}NO_4S$	15.0 g L^{-1}

[a)] only used for precultures in anaerobic shaken bottles, not in the stirred-tank bioreactor
